# Generating synthetic CT from low-dose cone-beam CT by using generative adversarial networks for adaptive radiotherapy

**DOI:** 10.1186/s13014-021-01928-w

**Published:** 2021-10-14

**Authors:** Liugang Gao, Kai Xie, Xiaojin Wu, Zhengda Lu, Chunying Li, Jiawei Sun, Tao Lin, Jianfeng Sui, Xinye Ni

**Affiliations:** 1 Radiotherapy Department, Second People’s Hospital of Changzhou, Nanjing Medical University, Changzhou, 213003 China; 2 Center for Medical Physics, Nanjing Medical University, Changzhou, 213003 China; 3 Oncology Department, Xuzhou No.1 People’s Hospital, Xuzhou, 221000 China; 4 School of Biomedical Engineering and Informatics, Nanjing Medical University, Nanjing, 213000 China

**Keywords:** Synthetic CT, Low-dose CBCT, Attention-guided GAN, Unpaired, Adaptive radiotherapy

## Abstract

**Objective:**

To develop high-quality synthetic CT (sCT) generation method from low-dose cone-beam CT (CBCT) images by using attention-guided generative adversarial networks (AGGAN) and apply these images to dose calculations in radiotherapy.

**Methods:**

The CBCT/planning CT images of 170 patients undergoing thoracic radiotherapy were used for training and testing. The CBCT images were scanned under a fast protocol with 50% less clinical projection frames compared with standard chest M20 protocol. Training with aligned paired images was performed using conditional adversarial networks (so-called pix2pix), and training with unpaired images was carried out with cycle-consistent adversarial networks (cycleGAN) and AGGAN, through which sCT images were generated. The image quality and Hounsfield unit (HU) value of the sCT images generated by the three neural networks were compared. The treatment plan was designed on CT and copied to sCT images to calculated dose distribution.

**Results:**

The image quality of sCT images by all the three methods are significantly improved compared with original CBCT images. The AGGAN achieves the best image quality in the testing patients with the smallest mean absolute error (MAE, 43.5 ± 6.69), largest structural similarity (SSIM, 93.7 ± 3.88) and peak signal-to-noise ratio (PSNR, 29.5 ± 2.36). The sCT images generated by all the three methods showed superior dose calculation accuracy with higher gamma passing rates compared with original CBCT image. The AGGAN offered the highest gamma passing rates (91.4 ± 3.26) under the strictest criteria of 1 mm/1% compared with other methods. In the phantom study, the sCT images generated by AGGAN demonstrated the best image quality and the highest dose calculation accuracy.

**Conclusions:**

High-quality sCT images were generated from low-dose thoracic CBCT images by using the proposed AGGAN through unpaired CBCT and CT images. The dose distribution could be calculated accurately based on sCT images in radiotherapy.

## Introduction

Cone-beam CT (CBCT) images are widely used in image-guided radiotherapy (IGRT) [1–3], and they are important for decreasing the positioning error and increasing the accuracy of treatments for patients with cancer. Compared with images from traditional fan-beam CT, CBCT images suffer from low contrast and artifacts due to X-ray scattering, mechanical accuracy, and movement of patients during scanning [4, 5], resulting in serious distortion of the Hounsfield unit (HU) value. Hence, CBCT images are unsuitable for calculating dose distributions for replanning in adaptive radiotherapy. Besides, patients may undergo multiple CBCT scans during an IGRT treatment course and this raises a great concern about delivered dose to the patients. Previous study indicated that daily CBCT scan for IGRT could increase the secondary cancer risk by 2% up to 4% [6]. To reduce the additional dose for patients generated from IGRT, the researchers have proposed several low-dose CBCT imaging technologies [7, 8]. Now, the low-dose protocols of CBCT scanning have been widely used in clinical practice.

Many methods of using CBCT images in adaptive radiotherapy have been proposed, and these include water–air–bone density assignment [9, 10], CBCT imaging process improvement based on modeling [11–13], and deformable image registration (DIR) of CT/CBCT images [14, 15]. Direct HU-ED calibration of CBCT images has relatively low accuracy due to the absence of artifact reduction processing. Electron density assignment is time consuming and influenced by human experience. Arai [16] modified the HU values of CBCT images to match the planning CT images by the histogram matching algorithm and evaluated in the phantom and head and neck cancer patients. Traditional model-based CBCT imaging correction is often realized by creating complex physical models to simulate the scattering [17–20] or changing of hardware. This method is difficult to promote due to limitations in hardware or calculation efficiency of physical models. Mainegra-Hing [17] calculated the scatter contribution of CBCT in the phantom by Monte Carlo (MC) algorithm. Niu [19] proposed a priori CT-based scatter correction method, where the corresponding planning CT projections were used to correct CBCT projections, and evaluated using two phantom studies. Park [20] applied the priori CT-based scatter correction technique to phantoms and a prostate patient for proton dose calculation. The priori CT-based scatter correction method is on the premise that the anatomical structure of CBCT is completely consistent with that of planning CT after registration which is difficult to satisfied in clinical practice such as the thorax and abdomen. DIR transforms planed CT to CBCT through deformation registration to account for anatomical changes. This type of method achieves good results at sites that are stationary, such as the head and neck. However, the method’s registration accuracy needs to be improved at sites with considerable anatomic structural changes, such as the chest and abdomen [15].

Another method of correcting the HU value of CBCT images is to generate synthetic CT (sCT) images from CBCT images through deep learning [21–31]. This method establishes a complicated mapping between CBCT and CT by training neural networks, thus allowing sCT images to be generated from CBCT directly. sCT has the same anatomical structure as CBCT, and the HU values of tissues are close to those of planning CT. Chen [22] used Unet to generate sCT images from the CBCT of patients with head and neck cancer, and the loss function combined the mean absolute error (MAE) and structural similarity index (SSIM). The MAE of sCT and CT in the testing results was 18.98 HU. Similarly, Li [21] added a residual unit to Unet to generate sCT from CBCT from patients with head and neck cancer, and the MAE between sCT and CT ranged within 6–27 HU. Instead of generating sCT directly, Hansen [28] proposed a ScatterNet where pairs of measured and corrected projections were trained using a Unet-like architecture. The corrected projection was obtained by the priori CT-based scatter correction method [19]. Lalonde [29] applied the MC simulation to generate CBCT projections for head and neck patients, then the Unet was trained to reproduce MC projection-based scatter correction from raw projections. The MAE of scatter-corrected images was 13.4 HU, compared to 69.6 HU for the uncorrected images. Landry [30] compared Unet training with three different datasets to correct CBCT images for prostate patients. The datasets include raw and corrected CBCT projections, raw CBCT image and DIR-synthetic CTs, raw CBCT image and reconstructed CBCT image based on corrected projections. Supervised learning methods, such as Unet [32], require paired CBCT/CT images as the training dataset, and voxel-wise loss is usually applied. However, these methods need high-accuracy alignment of paired images, which is difficult to acquire in clinics, especially at sites with considerable anatomical structural changes, such as the chest and abdomen.

The development of generative adversarial networks (GAN) [33] has provided a new technology and framework for the application of medical images. GAN has achieved state-of-the-art performance in many medical image tasks, including segmentation [34, 35], classification [36, 37] and medical image synthesis [38–40]. Isola[41] proposed conditional adversarial networks(cGAN) in image-to-image translation (so-called pix2pix) which was widely used in medical image reconstruction [40] and cross modality synthesis [38, 39]. Maspero [38] applied pix2pix in MR-to-sCT generation on 2D paired transverse image slices of 32 prostate cancer patients. Cusumano [39] used cGAN to generate sCT from low field MR(0.35 T) images in pelvis and abdomen for MR-guided adaptive radiotherapy. Quan [40] reconstructed the MR image form under-sampled K-space using pix2pix. Zhu [42] proposed an unsupervised cycle-consistent adversarial network (cycleGAN) to solve image translation for unpaired datasets, and it has been applied extensively in unpaired medical image translation [43]. Liang [23] utilized cycleGAN to generate sCT from the CBCT of patients with head and neck cancer by using an unpaired training dataset; a phantom experiment demonstrated that the method has better anatomical accuracy than the DIR method. Kida [26] conducted training on unpaired CBCT/CT images of 20 patients with prostate cancer by using cycleGAN and found that the image quality of sCT substantially improves compared with that of the original CBCT. Harms [24] fed 3D image patches to cycleGAN for CBCT-to-sCT image generation of patients with brain and pelvis cancer. They used paired CBCT and CT images in training and found that the mean absolute errors (MAEs) of sCT in the brain and pelvis are 13.0 and 16.1 HU, respectively. On the basis of the study of Harms [24], Liu [25] added self-attention to the generator network of cycleGAN for CBCT-to-sCT image generation of patients with pancreatic cancer and calculated the radiotherapy dose distribution. These studies on sCT generation from CBCT images concentrated on the head or abdomen, but limited studies have been conducted on CBCT images of the thorax, and low-dose CBCT-to-sCT image generation have not been studied.

In this study, unpaired low-dose CBCT and CT images of the thorax were trained using GAN. The low-dose CBCT images were obtained under a fast protocol with 50% less clinical projection frames compared with standard protocol. The sCT images generated from CBCT were used to calculate the dose distribution for adaptive radiotherapy. Given that the anatomical structure changes considerably due to respiratory movement, acquiring perfect alignment for CT/CBCT images is difficult. Hence, GAN was selected for unsupervised training. Furthermore, the low-dose CBCT images of the thorax include considerable artifacts, such as streaking, shading, and cupping caused by X-ray scatter and respiratory movements of patients; these artifacts disturb image translation tasks. We used attention-guided GAN (AGGAN) [44], which pays attention to the important part of images to eliminate numerous artifacts. Moreover, cycleGAN [42] and conditional GAN (so-called pix2pix) [41] were used in CBCT-to-sCT generation, and the quality of sCT images generated by different neural networks was compared. Then, a quantitative assessment of the generated sCT images was performed on a thoracic phantom, and the dose distribution of a radiotherapy plan was calculated.

## Materials and methods

### Image acquisition and processing

The low-dose CBCT and planning CT images of 170 patients who underwent free-breathing thoracic radiotherapy in our hospital were collected, 136 pairs as the training dataset and 34 pairs as testing dataset. The CBCT images were acquired through XVI scanning of a linear accelerator Infinity (Elekta, Stockholm, Sweden). In this study, a fast CBCT protocol was used for scanning to obtain low-dose CBCT images. Compared with the built-in standard protocol, a fast protocol accelerates the gantry rotation speed and decreases the scanning frames, thus decreasing the scanning time and radiation dose of patients. However, image quality is reduced to some extent [8, 45]. The fast protocol was realized by modifying the gantry rotation speed of the standard chest M20 protocol from 180°/min to 360°/min while the other parameters were kept constant. The projection frames were reduced from 660 to 330 for each patient scan. The gantry was rotated by 360° during each CBCT scanning, and 330 projection frames were collected. The planning CT images of patients were acquired using Siemens CT (SOMATOM Force, Germany). The scanning and reconstruction parameters of CBCT and CT are listed in Table1. The CT images were re-sampled to keep their resolution consistent with that of the CBCT images. Then, the CBCT images of each patient were used as fixed images, and the corresponding CT images were aligned with the CBCT images via 3D rigid registration. For the testing dataset, a deformable registration was performed on the CT to pair it to the corresponding CBCT by a multi-resolution B-spline algorithm. Afterward, the CT images were cropped to have the same field of view (FOV) as the corresponding CBCT images.

**Table 1:** The scanning and reconstruction parameters of CBCT and planning CT image

	Tube voltage (kVP)	Tube current (mA)	Spatial resolution (mm^2^)	Slice thickness (mm)	Image size
CBCT	120	20	1 × 1	2	410 × 410
Planning CT	120	220	0.97 × 0.97	2.5	512 × 512

### Image synthesis with AGGAN

**Fig. 1:**
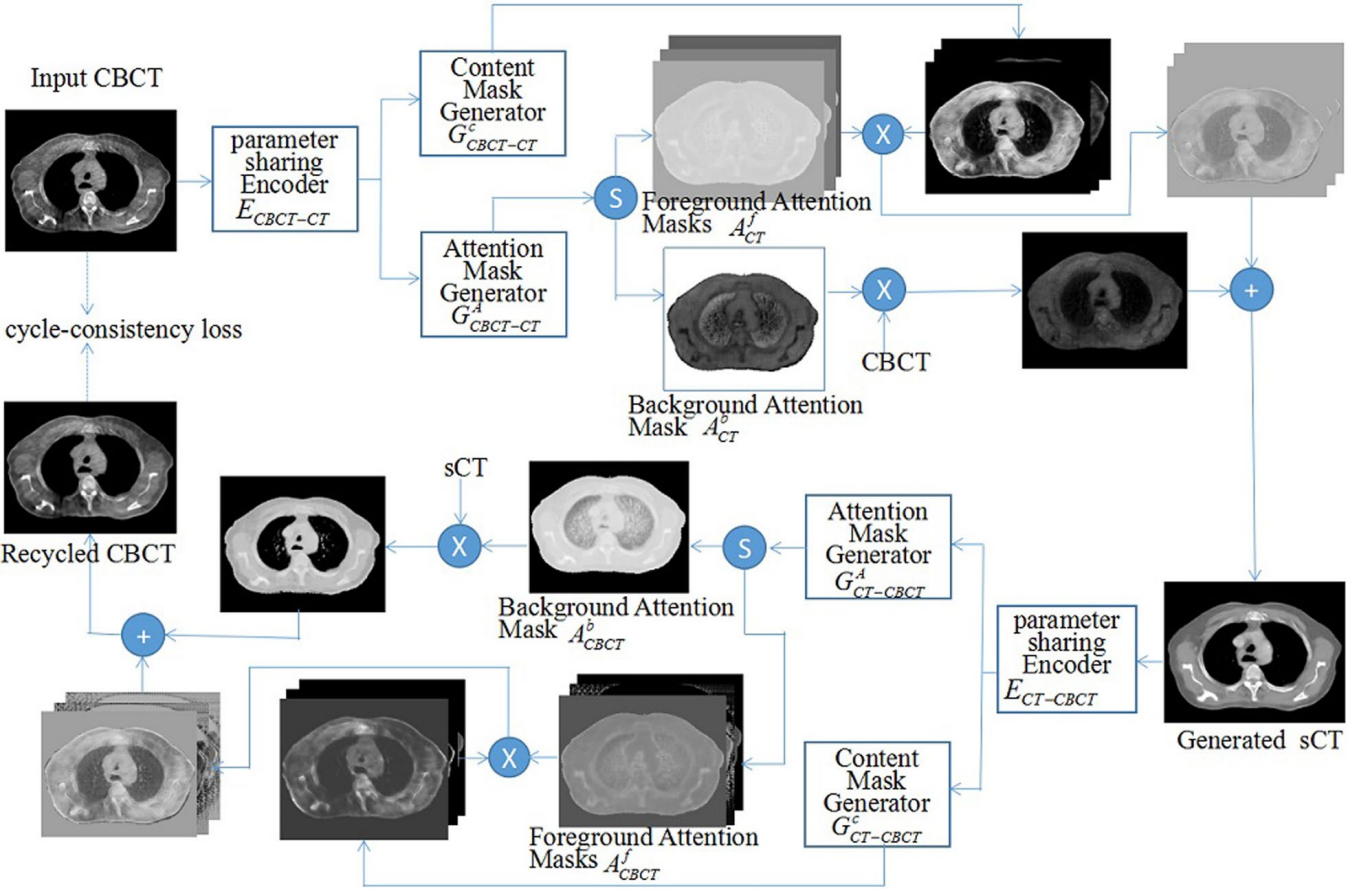
Framework of the proposed AGGAN, which contains two attention-guided generators *G*_*CBCT–CT*_ and *G*_*CT–CBCT*_. We show one cycle in this figure, i.e., CBCT → sCT → rCBCT ≈ CBCT. Each generator such as *G*_*CBCT–CT*_ consists of a parameter sharing encoder *E*_*CBCT–CT*_, a content mask generator $G_{CBCT - CT}^{C}$ and an attention mask generator $G_{CBCT - CT}^{A}$. The proposed model is constrained by the cycle-consistency loss. The symbols ⊕ ,  ⊗ and Ⓢ denote element-wise addition, element-wise multiplication and channel-wise Softmax respectively

AGGAN has a similar network structure as cycleGAN [44], and it involves two generators (*G*_*CBCT–CT*_ generates CT from CBCT and *G*_*CT–CBCT*_ generates CBCT from CT) and two discriminators (*D*_*CT*_ distinguishes the sCT from the real CT and *D*_*CBCT*_ distinguishes the synthetic CBCT (sCBCT) from real CBCT). AGGAN is composed of two cycles. In the first cycle, CBCT images are inputted into *G*_*CBCT–CT*_ to generate sCT images. Then, sCT images are inputted into *G*_*CT–CBCT*_ to generate recycled CBCT (rCBCT) images. The two discriminators distinguish the corresponding generative images. The cycle-consistency loss constrains the generation process to minimize the differences between the original CBCT images and the rCBCT images. In the second cycle, CT images are inputted into *G*_*CT–CBCT*_ to generate sCBCT images, which are then fed into *G*_*CBCT–CT*_ to generate recycled CT (rCT) images. Compared with the original cycleGAN, AGGAN modifies the generator network, which is equipped with a built-in attention module. A cycle process of AGGAN is shown in Fig. 1. *G*_*CBCT–CT*_ contains the encoding and decoding parts. The encoding part is a downsampling process that shares weights. The decoding part contains two branches; one generates n − 1 content masks, and the other generates n attention masks. The attention masks are divided into n − 1 foreground attention masks and one background attention mask after applying the Softmax function. $Soft\max (A_{i} ) = {{e^{{A_{i} }} } \mathord{\left/ {\vphantom {{e^{{A_{i} }} } {\sum\nolimits_{c = 1}^{n} {e^{{A_{c} }} } }}} \right. \kern-\nulldelimiterspace} {\sum\nolimits_{c = 1}^{n} {e^{{A_{c} }} } }}$, in which *A* is attention masks and *i* ranges from 1 to n. The background attention mask pays attention to the part that is unchanged before and after image generation, and this part is multiplied with the input CBCT images to obtain an output image. The foreground attention mask pays attention to the image part that changes before and after generation. A total of n − 1 output images are obtained by element-wise multiplication of n − 1 foreground attention masks and n − 1 content masks. These n output images are added, thus obtaining the final sCT images.

The *G*_*CBCT–CT*_ of AGGAN generates sCT images through Eq. (1).

1 $$S_{CT} = \sum\limits_{f = 1}^{n - 1} {(C_{CT} * A_{CT}^{f} )} + I_{CBCT} * A_{CT}^{b}$$

where *C*_*CT*_ is the content mask, $A_{CT}^{f}$ is the foreground attention mask, $A_{CT}^{b}$ is the background attention mask, *I*_*CBCT*_ is the input CBCT images, and *S*_*CT*_ is the generated sCT images. In this study, n was set to 10.

The generator *G*_*CT–CBCT*_ generates rCBCT images through Eq. (2).

2 $$R_{CBCT} = \sum\limits_{f = 1}^{n - 1} {(C_{CBCT} * A_{CBCT}^{f} )} + S_{CT} *A_{CBCT}^{b}$$

Corresponding to the variables in Eq. (1), *C*_*CBCT*_, $A_{CBCT}^{f}$, and $A_{CBCT}^{b}$ in Eq. (2) denote the content, foreground attention, and background attention masks in *G*_*CT–CBCT*_, respectively. *S*_*CT*_ is the sCT images gained from Eq. (1), and *R*_*CBCT*_ is the generated rCBCT images.

The adversarial loss of the neural network uses the LSGAN (Least Squares GAN) model [46] as shown in Eq. (3) and (4). *D*_*CT*_ distinguishes sCT from CT and aims to classify sCT with label 0 from CT with label 1. Differently, *G*_*CBCT–CT*_ attempts to make sCT as close as possible to CT and aims to output 1 for sCT after the discriminator. The loss functions of the discriminators and generators are the minimum and $L_{{GAN - G_{CBCT - CT} }}$, respectively.

3 $$L_{{GAN - D_{CT} }} = \, \frac{{1}}{2m}\sum\limits_{i = 1}^{m} {[(D_{CT} (I_{{_{CT} }}^{i} ) - 1} )^{2} + D_{CT} (G_{CBCT - CT} (I_{{_{CBCT} }}^{i} ))^{2} ]$$

4 $$L_{{GAN - G_{CBCT - CT} }} = \, \frac{{1}}{2m}\sum\limits_{i = 1}^{m} {(D_{CT} (G_{CBCT - CT} (I_{{_{CBCT} }}^{i} ) - 1} ))^{2}$$

where *m* is the number of trained images and and $I_{CBCT}^{i}$ are the *i*th CT and *i*th CBCT images, respectively. The loss functions of *D*_*CBCT*_ and *G*_*CT–CBCT*_ are similar to those in Eqs. (3) and (4).

5 $$L_{{GAN - D_{CBCT} }} = \, \frac{{1}}{2m}\sum\limits_{i = 1}^{m} {[(D_{CBCT} (I_{{_{CBCT} }}^{i} ) - 1} )^{2} + D_{CBCT} (G_{CT - CBCT} (I_{{_{CT} }}^{i} ))^{2} ]$$

6 $$L_{{GAN - G_{CT - CBCT} }} = \, \frac{{1}}{2m}\sum\limits_{i = 1}^{m} {(D_{CBCT} (G_{CT - CBCT} (I_{{_{CT} }}^{i} ) - 1} ))^{2}$$

The generative adversarial loss is

7 $$L_{GAN} = L_{{GAN - D_{CT} }} + L_{{GAN - G_{CBCT - CT} }} + L_{{GAN - D_{CBCT} }} + L_{{GAN - G_{CT - CBCT} }}$$

The neural network still can map images from one domain to several domains on the basis of generative adversarial loss only. These domains share the same distribution characteristics, which cannot ensure that the learned generator can map the input CBCT images to the desired CT images. A cycle-consistency loss needs to be added to decrease the mapping function spaces as much as possible; this loss requires the minimum difference between the input CBCT and rCBCT images and the minimum difference between the input CT and rCT images.

8 $$L_{cycle - CBCT} = \, \frac{{1}}{m}\sum\limits_{i = 1}^{m} {\left| {G_{CT - CBCT} (G_{CBCT - CT} (I_{{_{CBCT} }}^{i} )) - I_{{_{CBCT} }}^{i} } \right|}$$

9 $$L_{cycle - CT} = \, \frac{{1}}{m}\sum\limits_{i = 1}^{m} {\left| {G_{CBCT - CT} (G_{CT - CBCT} (I_{{_{CT} }}^{i} )) - I_{{_{CT} }}^{i} } \right|}$$

10 $$L_{cycle} = L_{cycle - CBCT} + L_{cycle - CT}$$

*G*_*CBCT–CT*_ generates CT images from the input CBCT images. If CT images are inputted into *G*_*CBCT–CT*_, then the difference between the generated CT image and the input CT images should be small as possible, and it will be constrained by identity loss.

11 $$L_{idt - CT} = \, \frac{{1}}{m}\sum\limits_{i = 1}^{m} {\left| {G_{CBCT - CT} (I_{{_{CT} }}^{i} ) - I_{{_{CT} }}^{i} } \right|}$$

12 $$L_{idt - CBCT} = \, \frac{{1}}{m}\sum\limits_{i = 1}^{m} {\left| {G_{CT - CBCT} (I_{{_{CBCT} }}^{i} ) - I_{{_{CBCT} }}^{i} } \right|}$$

13 $$L_{idt} = L_{idt - CT} + L_{idt - CBCT}$$

The total loss function is the sum of the three loss functions.

14 $$L = L_{GAN} + \lambda_{cycle} * L_{cycle} + \lambda_{idt} * L_{idt}$$

In the experiment, *λ*_*cycle*_ was set 10, and *λ*_*idt*_ was set 5.

### Neural network training

In conventional thoracic CT images, a few pixels have HU > 1500. In this study, the HU value of images was clipped to [− 1000, 1500] HU, and those exceeding 1500 HU were set to 1500 HU. Afterward, the pixel values were scaled to [− 1, 1] and inputted into the neural network. In consideration of the requirements on GPU memory and training efficiency of the neural network, 2D axial slices of the CT images were used and resized to 256 × 256 for training. Kida [26] pointed out that 2D axial slices of CT images can generate good sCT images and do not cause structural discontinuity in coronal and sagittal views. The training dataset contained thoracic CT and CBCT images of 136 patients and involved 12,784 slices of CBCT and CT images. The testing dataset contained 3,196 slices of CBCT images of 34 patients. During the training of AGGAN and cycleGAN, the CBCT and CT images were shuffled in each epoch so that they could be trained through unpaired data. The pix2pix network was trained using the paired CBCT and CT images.

In AGGAN, the downsampling of the generator consisted of (a) one convolution layer with a 7 × 7 kernel with a stride of 1 and 64 channels, (b) two convolution layers with a 3 × 3 kernel with a stride of 2 and 128,256 channels, and (c) 9 residual blocks with a 3 × 3 kernel with a stride of 1 and 64 channels. The upsampling involved two independent branches of content and attention masks. The first of the two branches had two deconvolution layers with a 3 × 3 kernel with a stride of 2 and 128, 64 channels using the ConvTranspose2d function. The last layer of the content mask was a 7 × 7 convolution layer with a stride of 1 and 9 channels. The last layer of the attention mask was a 1 × 1 convolution layer with a stride of 1 and 10 channels. Instance normalization was performed after each convolution layer except for the last layer, and ReLU was used as the activation function [47]. The discriminator used PatchGAN in pix2pix [41], the mean of all patches in an image was calculated to judge whether the entire image was true or false. The batch size was set to 1 during training, and the Adam optimizer was used for optimization. The momentum was set to β1 = 0.5 and β2 = 0.999, and a total of 100 epochs were established. The initial learning rate of Adam was set to 0.0001, and after 50 epochs the learning rate started linearly decaying to 0. Pix2pix [41] and cycleGAN [42] were trained in the way indicated in the original paper, and the number of epochs was set to 100. The neural networks were implemented in the PyTorch framework with Pycharm software and the training was done on a NVIDIA 2080 Ti Graphical Processing Unit(GPU). The training computation time for the pix2pix, cycleGAN and AGGAN was 428 h, 655 h, 732 h respectively. Once trained, the network is able to generate sCT from CBCT images with mean speed of 141 slices/min, 142 slices/min and 133 slices/min for the pix2pix, cycleGAN and AGGAN respectively. That is to say the trained networks can generate sCT for a patient (usually less than 100 slices) within one minute.

### Evaluation

A side-by-side comparison of true CT images, CBCT and sCT generated by pix2pix, cycleGAN, AGGAN was performed at the mediastinal window of [− 400, 400] HU and lung window of [− 1200 300] HU. The HU histogram distribution of one patient’s 3D images were also compared. The sCT images of patients generated from neural networks in testing dataset were quantitatively evaluated by computing the MAE, SSIM, and peak signal-to-noise ratio (PSNR) with deformed CT images as the reference. An intensity-modulated radiation therapy phantom (002LFC, CIRS, USA) was scanned by CT and CBCT. The CBCT and CT images of the phantom were aligned through 3D rigid registration. The image quality of the sCT images for phantom was quantitatively evaluated using the CT images as reference. In addition, three regions of interest (ROI) were identified on each image (lung, bone and soft tissues). The mean HU values with standard deviation (SD) of ROIs for test patients or phantom were calculated and compared. The image quality indices were compared by paired Wilcoxon rank test and the statistical significance level was set at *P* < 0.05.

To verify the dose calculation accuracy, the treatment plans of the 34 test patients were copied to deformed CT images, CBCT and sCT images in the treatment planning system (Monaco5.1, Elekta). The dose distribution were calculated directly without optimized in these images. Using the dose distribution of deformed CT images as reference, the 3D gamma passing rates of the dose distributions on CBCT and sCT images were calculated under different criteria (distance to agreement and relative dose difference). In addition, a treatment plan was designed based on the phantom to simulate lung cancer radiotherapy. Volumetric-modulated arc therapy (VMAT) was adopted. The radiation field was rotated by 360°, and target 5000 cGy of the prescription dose was applied. Subsequently, dose distribution was calculated and compared in CT, CBCT and sCT images of the phantom.

## Results

### Comparison of image quality and preservation of the anatomical structure

**Fig. 2:**
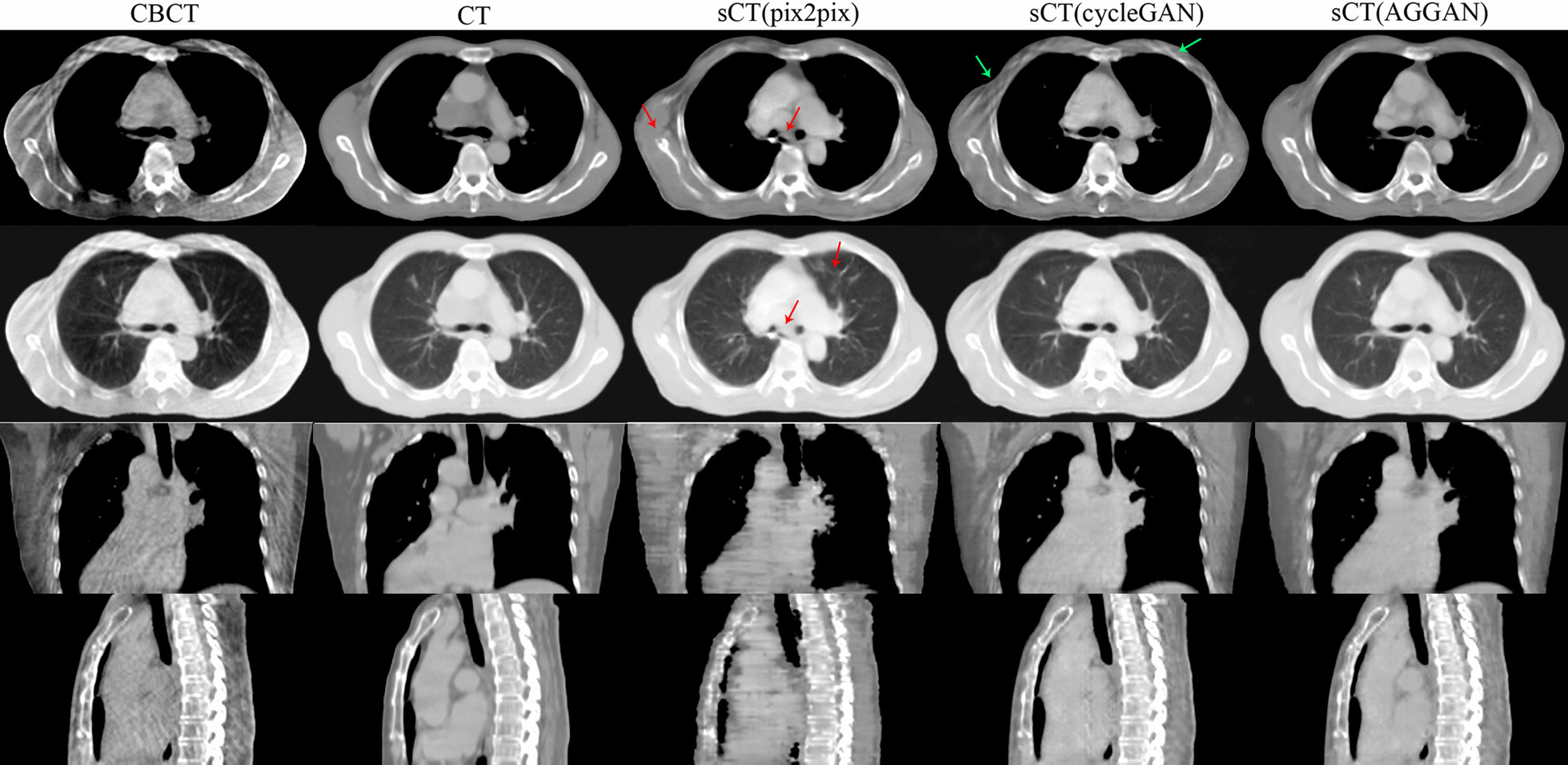
Quality comparison of CBCT, CT, and sCT images generated by three neural networks for the same patient in axial, coronal, and sagittal images. The display window in second row is [− 1200 300] HU (lung window), and display window in other rows is [− 400 400] HU

The sCT images generated by different neural networks from the CBCT of the same patient in the test are shown in Fig. 2. Each row shows images of the same slice. From top to bottom, the rows denote axial slices of the mediastinal window display, axial slices of the lung window display, and coronal and sagittal images. The first and second rows show the same slice. The columns from left to right display CBCT, CT, and sCT images generated by pix2pix, cycleGAN, and AGGAN, respectively. Serious streaking and shading artifacts were observed at the chest wall and other sites of the original CBCT images, respectively, due to the influence of the respiratory movement of patients during scanning. The lung window shows that the lung was relatively dark, and the HU value in CBCT had serious distortion. Most of the artifacts in the sCT images generated by pix2pix were eliminated, but several anatomical structures, especially the bone, cavity, and lung marking regions (red arrows), in the images were destroyed. The coronal and sagittal images presented serious image distortions. The sCT images generated by cycleGAN maintained several streaking artifacts on the axial slices. In particular, the chest wall (green arrow in the images) had serious artifacts, but the anatomical structure was preserved well. The coronal and sagittal images revealed good tissue continuity. Most artifacts in the sCT images generated by AGGAN were eliminated, and the anatomical structures were well-preserved. The quality of the coronal and sagittal images was also improved.

**Fig. 3:**
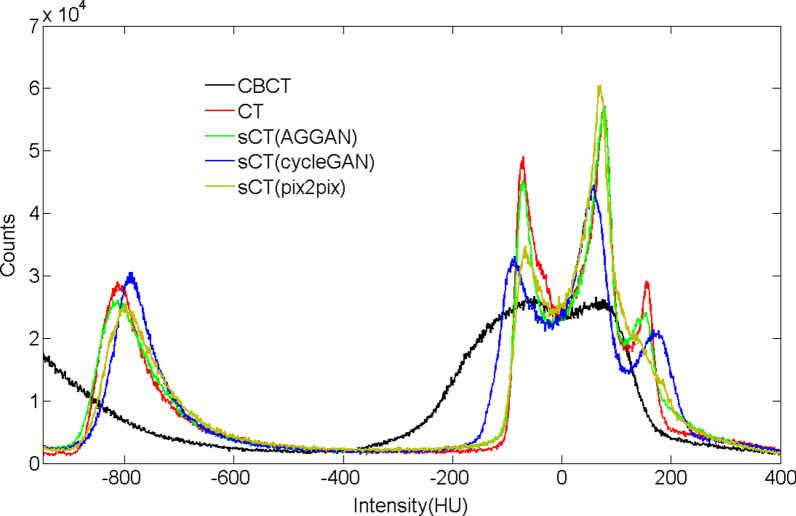
Histogram distribution curves of the HU values of 3D CT, CBCT, and sCT images generated by three neural networks

The image histograms of the 3D images for patients shown in Fig. 2 were analyzed (Fig. 3). In Fig. 3, the x-axis denotes the HU value, and the y-axis denotes the number of occurrences of HU values in the 3D CT images. The HU value distributions of the CBCT and CT images differed considerably. The HU value of the sCT images generated by neural networks showed a similar distribution as that of the real CT images. The distribution curves of the CT and sCT images had an evident peak at about − 800 HU, which is the HU value distribution of the lung. However, such a peak was absent in the CBCT images. The HU value distribution of the sCT images generated by AGGAN was the closest to that of the CT images.

**Fig. 4:**
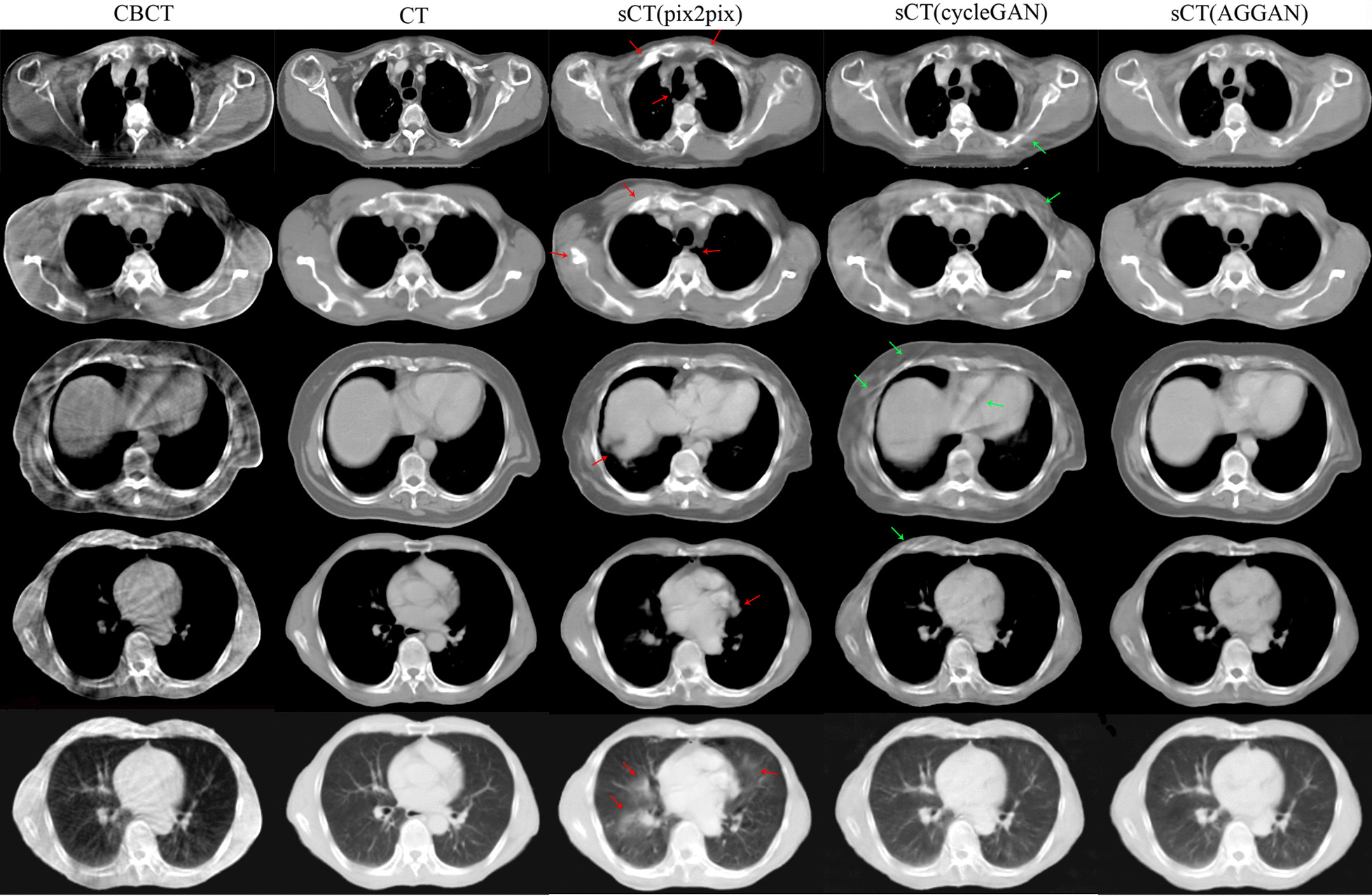
Comparison of sCT images generated by the three neural networks from CBCT images of patients in the test. The display window in bottom row is [− 1200 300] HU (lung window), and display window in other rows is [− 400 400] HU

**Fig. 5:**
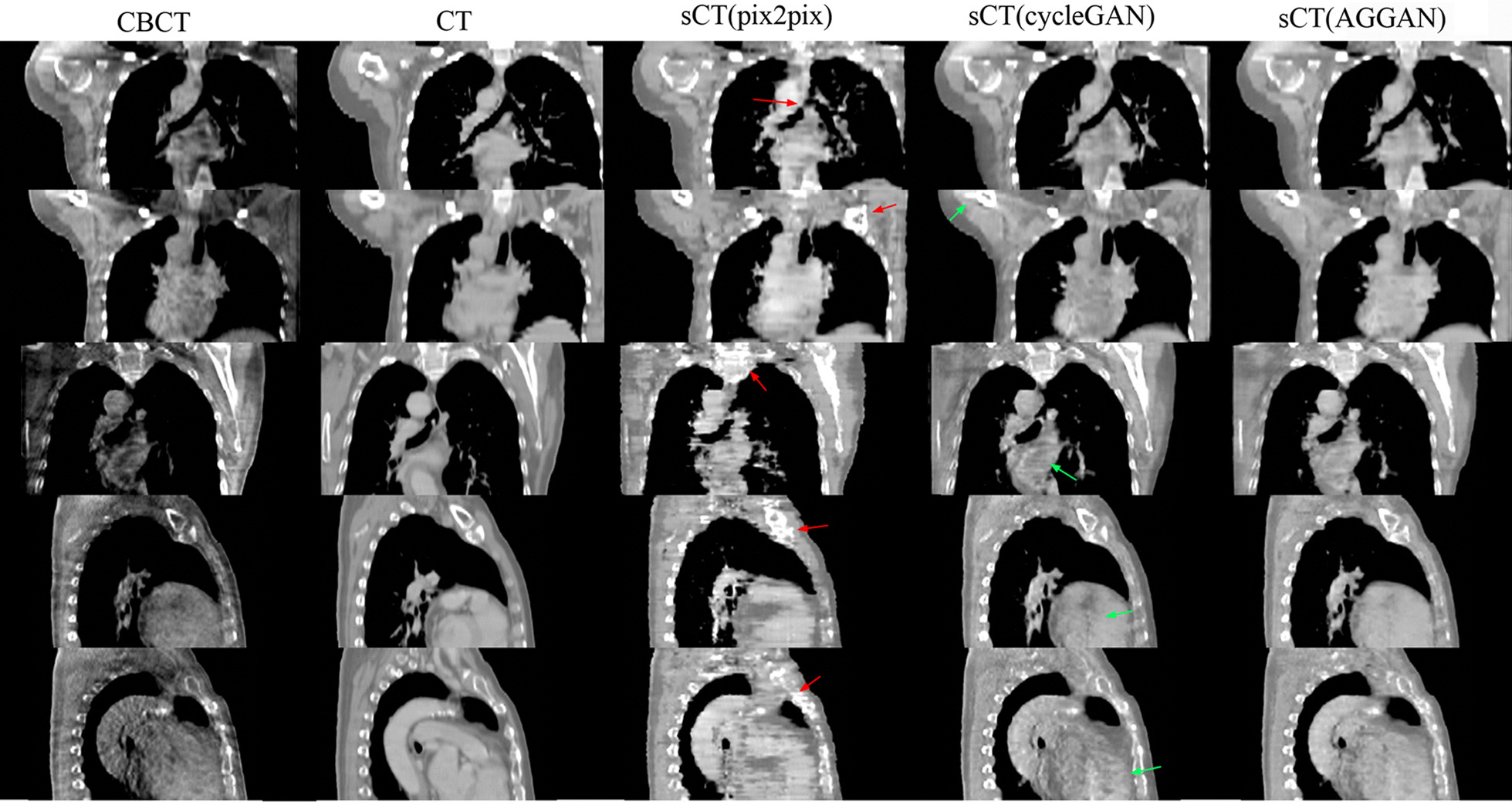
Comparison of sCT images generated by the three neural networks from test CBCT images in coronal (the top three rows) and sagittal (the bottom two rows) views. The display window is [− 400 400] HU

The sCT images generated from the CBCT images of test patients in axial view are shown in Fig. 4. The first four rows from the top to the bottom are axial slice images of different patients, and the fifth row is the lung window display of the fourth row. The same row shows the same slice of images of patients. The generated sCT images in coronal and sagittal views are shown in Fig. 5. Similar to the results in Fig. 2, the CBCT images included many artifacts, and the HU value had serious distortion. Pix2pix eliminated many artifacts but destroyed the anatomical structures, which mainly included bone tissues, cavities, lung marking, liver, and heart edges (red arrows). The sCT images generated by cycleGAN had well-preserved anatomical structures but retained several streaking artifacts from the CBCT images (green arrow). The sCT images generated by AGGAN reduced more artifacts than cycleGAN, and preserved the anatomical structures well.

The quantitative analysis results of CBCT and sCT on image quality for testing patients are listed in Table 2. The image quality indices of all sCT images are significantly improved compared with original CBCT images (*P* < 0.05). The AGGAN achieves the best image quality with the smallest MAE, largest SSIM and PSNR. The image quality indices of sCT generated from cycleGAN and AGGAN are both better than pix2pix. Compared with cycleGAN, sCT images generated from AGGAN show significant superiority in MAE and PSNR (*P* < 0.05). There are no significant difference in SSIM between sCT images generated from cycleGAN and AGGAN (*P* = 0.261). The sCT images of patients generated by AGGAN show the best image quality. The mean HU values of ROIs on CT, CBCT and sCT images for patients are listed in Table 3. The mean HU values of lung, bone and soft tissue on CBCT images are significantly less than that on CT images (*P* < *0.05*). In addition, the mean HU values of ROIs on CBCT images fluctuated greatly, leading to a large SD number. The mean HU values of ROIs on sCT images generated from three networks are close to that on CT images. There are no significant difference on mean HU values of ROIs among CT, sCT generated from pix2pix, cycleGAN and AGGAN.

**Table 2:** Image quality indices of CBCT and sCT images generated by the three neural networks

	CBCT	sCT (pix2pix)	sCT (cycleGAN)	sCT (AGGAN)
MAE (HU)	92.8 ± 16.7	53.4 ± 9.34	47.1 ± 6.45	43.5 ± 6.69
SSIM (%)	78.3 ± 6.34	88.1 ± 7.12	93.2 ± 4.17	93.7 ± 3.88
PSNR (dB)	21.6 ± 2.81	26.8 ± 2.73	28.3 ± 2.04	29.5 ± 2.36

**Table 3:** The mean HU values of ROIs on CT, CBCT and sCT images for patients

	Mean HU value of ROIs (mean ± SD)				
	CT	CBCT	sCT (pix2pix)	sCT (cycleGAN)	sCT (AGGAN)
Lung (HU)	− 722.6 ± 46.1	− 853.9 ± 85.2	− 708.2 ± 49.4	− 704.8 ± 40.8	− 710.3 ± 42.7
Bone (HU)	217.4 ± 23.8	67.1 ± 42.7	223.0 ± 27.5	208.7 ± 25.1	211.7 ± 27.2
Soft tissue (HU)	13.7 ± 16.2	− 116.7 ± 75.0	14.2 ± 15.1	9.6 ± 14.7	12.4 ± 15.7

### Dose calculation

**Fig. 6:**
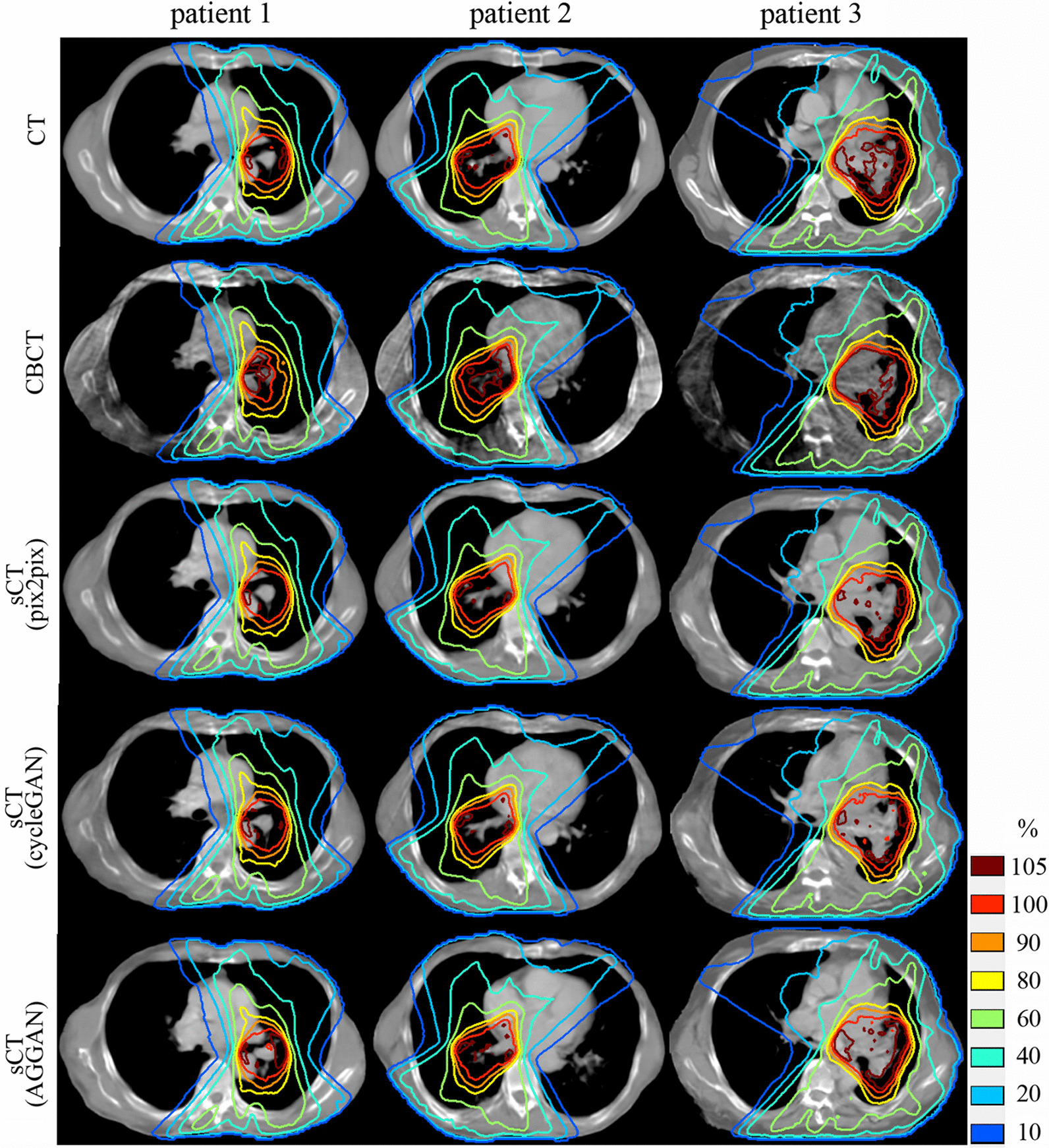
The relative dose distribution calculated on CT, original CBCT and generated sCT images

**Fig. 7:**
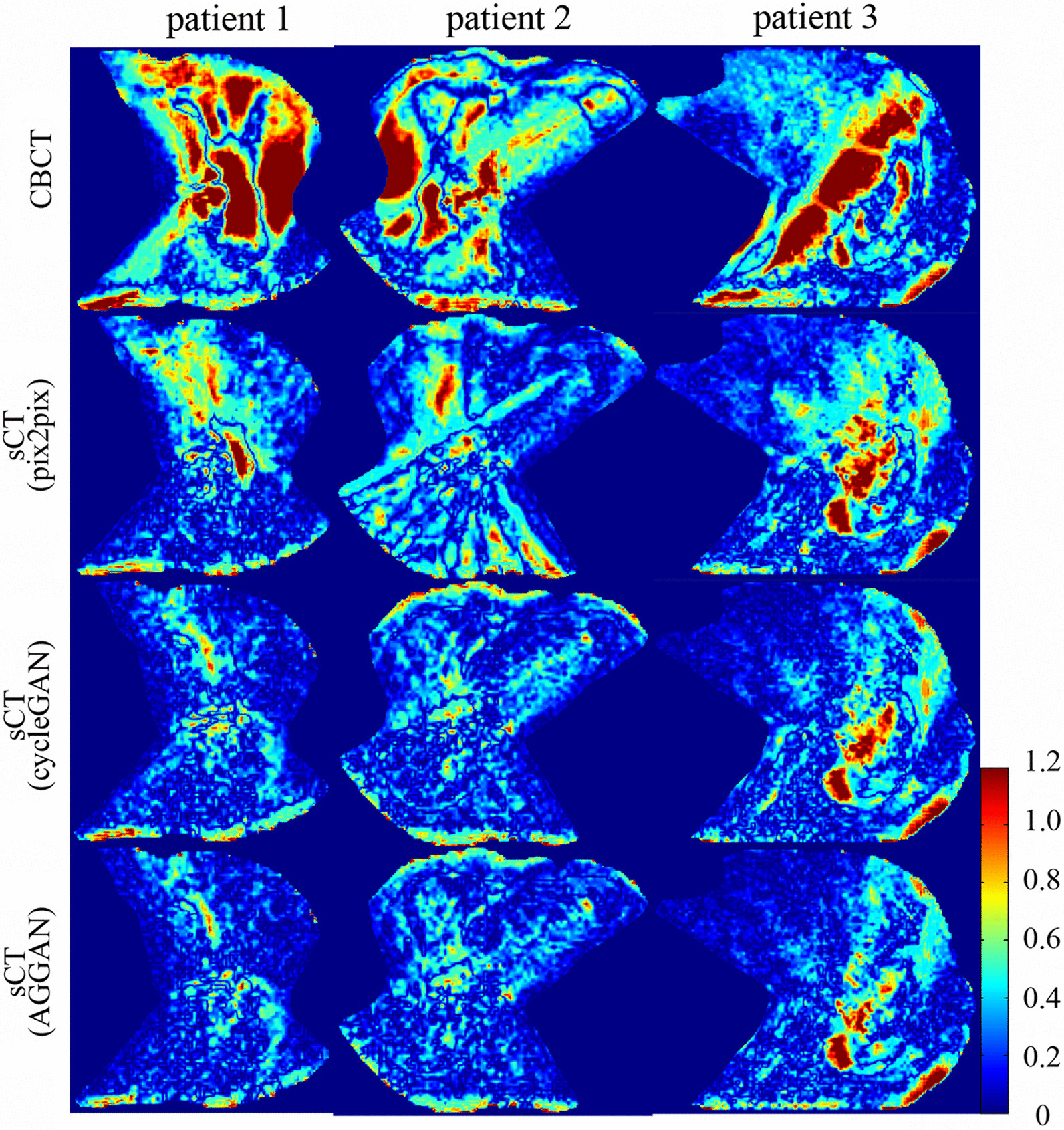
The gamma analysis index distribution calculated on original CBCT and generated sCT images with dose on CT image as reference under the criteria 2 mm/2%

The relative dose distribution calculated in treatment plans on CT, CBCT and sCT images for patients are shown in Fig. 6. Using the dose distribution calculated in the CT images as a reference, the absolute gamma analysis distribution of each corresponding image under the criteria 2 mm/2% are shown in Fig. 7. The dose distributions in the original CBCT images remained highly divergent compared with the reference. There are large regions where the gamma index is greater than 1 in CBCT images. The dose distribution of the sCT images are close to the reference, and the areas with a gamma index greater than 1 are greatly reduced. The statistical analysis of 3D gamma passing rates with different standards for the 34 testing patients are shown in Table 4. The gamma passing rates of sCT images generated from three methods were significantly improved under all criteria compared with that of original CBCT (*P* < 0.05). Under the criteria 1 mm/1% and 2 mm/2%, the gamma passing rates of cycleGAN and AGGAN were significantly increased compared with that of pix2pix (*P* < 0.05), but no significant differences were observed under 3 mm/3% criteria (*P* = 0.165). There are no significant differences for the gamma passing rates between cycleGAN and AGGAN under criteria 2 mm/2% or 3 mm/3% (*P* = 0.214 and *P* = 0.345). However, AGGAN got significantly higher passing rates than cycleGAN under the 1 mm/1% criteria (*P* < 0.05). In conclusion, the SCT images generated by AGGAN obtained the optimal dose calculation accuracy in radiotherapy for testing patients.

**Table 4:** The 3D gamma passing rates of dose distribution in CBCT and sCT images for patients

	Gamma criteria		
	1 mm/1%	2 mm/2%	3 mm/3%
CBCT (%)	50.1 ± 9.04	84.4 ± 5.81	92.8 ± 3.86
sCT-pix2pix (%)	75.3 ± 4.50	96.7 ± 2.26	99.4 ± 0.67
sCT-cycleGAN (%)	89.3 ± 3.81	98.2 ± 2.09	99.7 ± 0.44
sCT-AGGAN (%)	91.4 ± 3.26	98.6 ± 1.78	99.7 ± 0.39

### A phantom study

**Fig. 8:**
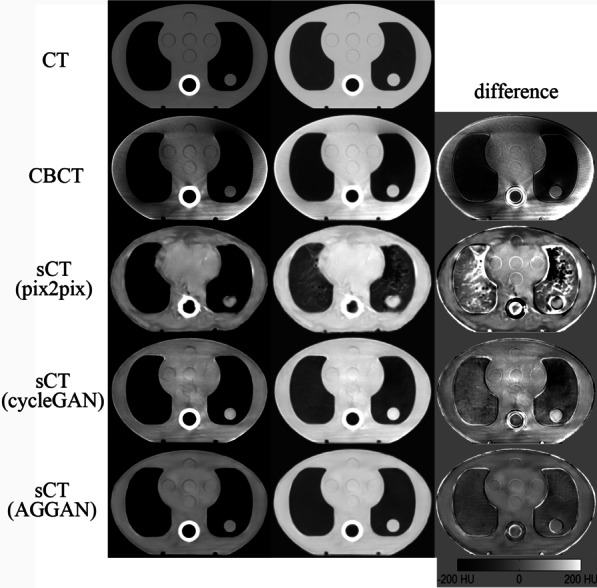
Quality comparison of CT, CBCT, and sCT images generated by the three neural networks. The display window in left column is [− 400 400] HU, and in middle column is [− 1200 300] HU

**Fig. 9:**
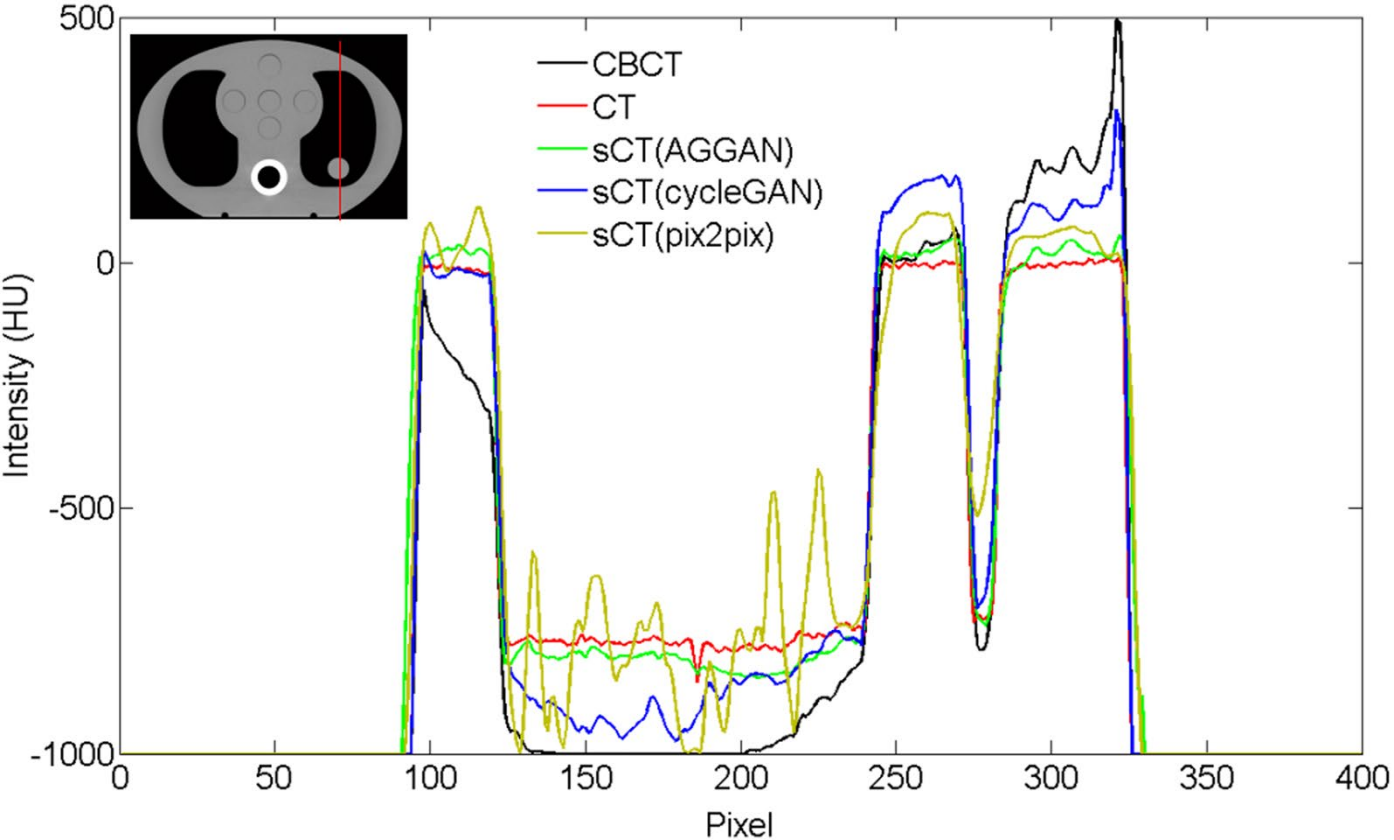
HU value distribution of images on the red straight

The CT, CBCT, and sCT images of the phantom are shown in Fig. 8. The images from the left to the right are the soft tissue window display, lung window display, and the image difference. The image difference was obtained by subtracting the CT image from each image. The dark region represents the HU value of the image part that is lower than that of the CT images, and the bright region represents the HU value of the image that is higher than that of the CT images. The original CBCT image of the phantom had large differences with the CT images. The lung tissues were relatively dark, and the soft tissue regions showed irregular shading. The sCT image generated by pix2pix destroyed the original structures seriously. However, the sCT images generated by cycleGAN and AGGAN retained the structures of the phantom well. The sCT images generated by cycleGAN showed a dark region at the right side of the lung, and the HU value of the cylinder inserted into the lung was larger than that in CT. The sCT images generated by AGGAN showed no large differences. The HU profile on one straight (red straight) of images is shown in Fig. 9, where considerable differences can be observed between the CBCT and CT images. The HU value of the CBCT images in the lung was close to zero. The HU value distribution of the sCT images generated by AGGAN was the closest to that of the CT images.

The CT images of the phantom were used as the reference, and the MAE, SSIM, and PSNR of the different images were calculated. The results are listed in Table 5. The MAE of the sCT generated by AGGAN was the lowest (23.2 HU), but its SSIM and PSNR were the highest (0.944 and 30.2, respectively). The SSIM (0.938) of the sCT images generated by cycleGAN was close to that of AGGAN, but the MAE (32.6 HU) was higher. Pix2pix exerted a poor experimental effect on the phantom, as manifested by the lower SSIM and PSNR than those of the original CBCT images. The MAE of pix2pix was hardly improved compared with that of the CBCT images. In addition, the lung, bone and soft tissue were contoured and the mean HU value of these ROIs were calculated, as shown in Table 6. The mean HU value of sCT generated by AGGAN are closest to that of CT on three ROIs. In the phantom experiment, the sCT images generated by AGGAN showed the best quality.

**Table 5:** Image quality indices of CBCT and sCT images in the phantom

	CBCT	sCT (pix2pix)	sCT (cycleGAN)	sCT (AGGAN)
MAE(HU)	62.9	62.3	32.6	23.2
SSIM	0.842	0.828	0.938	0.944
PSNR	25.8	24.0	28.5	30.2

**Table 6:** The mean HU values of ROIs on CT, CBCT and sCT images for phantom

	Mean HU value of organs (mean ± SD)				
	CT	CBCT	sCT (pix2pix)	sCT (cycleGAN)	sCT (AGGAN)
Lung(HU)	− 755.0	− 899.7	− 665.2	− 776.8	− 763.5
Bone (HU)	716.2	964.7	570.9	802.0	673.4
Soft tissue (HU)	− 27.5	10.3	44.1	24.3	− 6.8

**Fig. 10:**
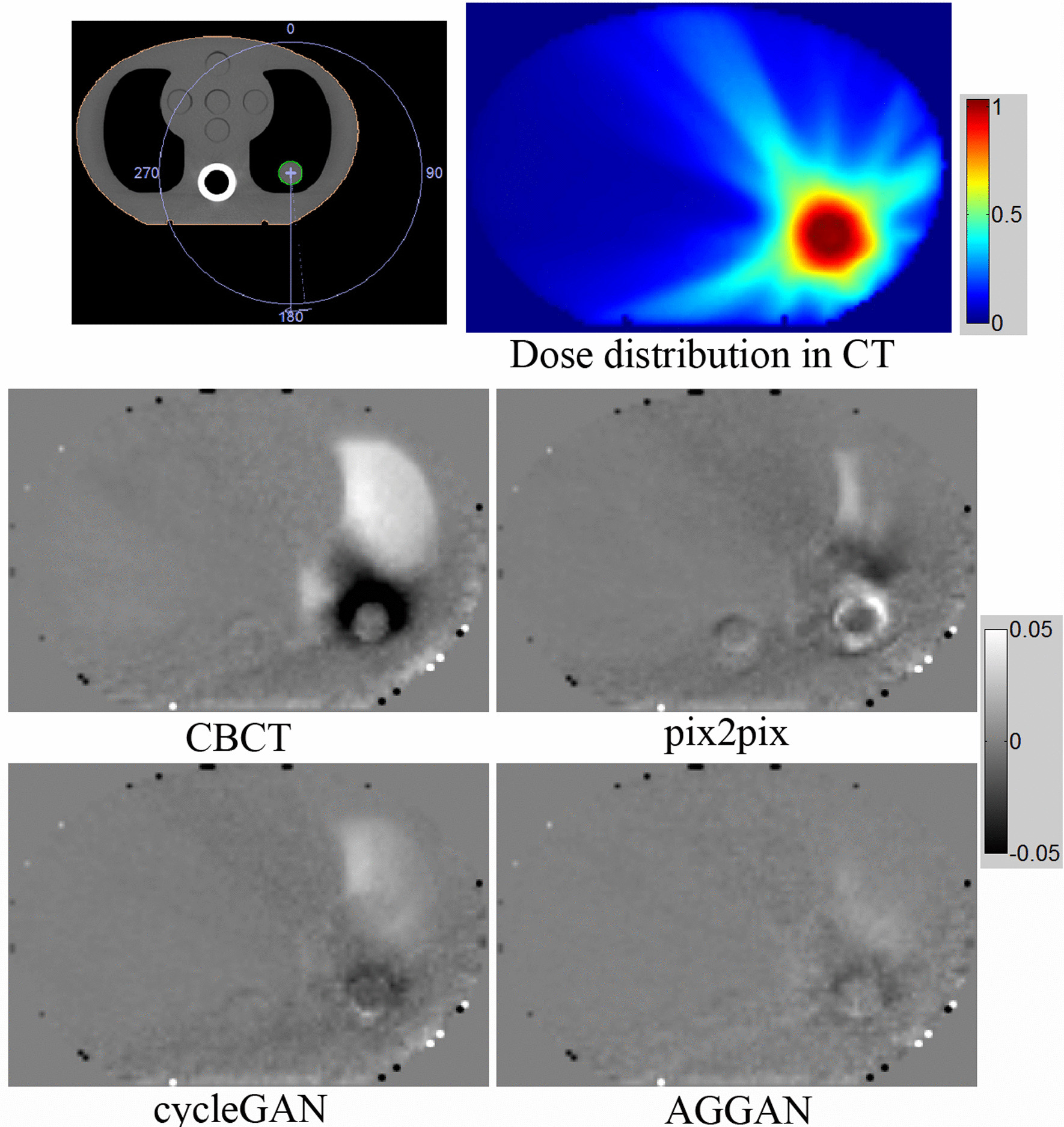
Dose distribution in CT images and distributions of relative dose differences in CBCT and sCT images genarated from pix2pix, cycleGAN and AGGAN

The calculated dose distribution in the phantom is shown in Fig. 10. The upper left part presents diagrams of the irradiation field and the target area (green profile), and the upper right part presents the calculated dose distribution in the CT images. The lower left and lower right parts show the relative dose difference distributions calculated on CBCT and sCT images generated from pix2pix, cycleGAN, AGGAN respectively. The relative dose difference distribution was obtained through the calculated dose from CBCT or sCT images minus the dose in the CT images and divided by the maximum dose in the CT images. Dark regions indicate that the calculated dose is lower than the reference dose, and the bright regions indicate that the calculated dose is higher than the reference dose. The high-dose regions (close to the target) in the CBCT images had large differences compared with CT. The dose difference in the sCT images reduced and minimum in AGGAN. The 3D gamma passing rates of the dose distributions on CBCT and sCT images with different standards were calculated (Table 7). The passing rates of dose distributions on the sCT images were higher than those on the CBCT images under all standards. Given the strictest standard of 1 mm/1%, the passing rate of the sCT images generated from AGGAN reached as high as 96.5%, but that of the CBCT images was only 79.8%. sCT images generated by AGGAN are thus conducive to calculating radiotherapy doses accurately.

**Table 7:** 3D gamma passing rates of dose distribution on CBCT and sCT images in the phantom

	Gamma criteria		
	1 mm/1%	2 mm/2%	3 mm/3%
CBCT (%)	79.8	91.6	96.4
sCT-pix2pix (%)	86.4	97.8	99.2
sCT-cycleGAN (%)	91.1	99.3	100
sCT-AGGAN (%)	96.5	99.9	100

## Discussions

In this study, sCT images were generated from low-dose CBCT images of thoracic patients by using pix2pix, cycleGAN, and AGGAN. The paired datasets were used in pix2pix training, whereas cycleGAN and AGGAN applied unpaired training datasets. The pix2pix reduced most of the artifacts of the original CBCT images in axial slices, but it destroyed the anatomical structures of normal tissues, resulting in image ambiguity and structural discontinuity in sagittal and coronal images. In the phantom study, pix2pix exerted great structural damages and failed to improve the image quality. The poor test results of pix2pix may be attributed to the incomplete alignment between CBCT and CT images in the training dataset. In this study, the training dataset was obtained through 3D rigid registration of CT and CBCT images. The CT and CBCT images after registration had evident local mismatching resulting from anatomical structure changes and movement of organs during the two scanning events. In particular, tissue structures, such as the trachea, esophagus, and bones, and the organ water/air filling status did not correspond to one another. Li [21] and Chen [22] implemented paired training by the Unet structure and generated sCT images based on CBCT images of patients with head and neck cancer. Given that organs in the head and neck are stationary, a good training dataset was obtained after the registration of CBCT and CT images. Liu [25] used a paired training dataset through DIR of CBCT and CT images of patients with pancreatic cancer. The images were collected from patients who received stereotactic body radiation therapy and held their breath, and relatively small differences among images were obtained. In conventional radiotherapy, thoracic CBCT images have serious artifacts due to respiration movement, and accurate DIR with CT images is facing a great challenge [15]. Liang [23] conducted a phantom study of the head and neck and proved that sCT images generated by neural networks have a more accurate anatomical structure than CT images obtained from DIR. Supervised learning methods, such as pix2pix, can only generate high-quality sCT images under the premise of accurate alignment between CBCT and CT images. Unsupervised learning methods, such as cycleGAN, do not depend on image registration results; thus, the generated sCT images maintain the anatomical structures well, and sagittal and coronal images have continuous structures. This finding is similar to the result of Liang [23] for head and neck images. However, the sCT images generated by cycleGAN in our experiment retained several artifacts. Given that the thoracic CBCT images contained more artifacts than the CBCT images of the head and neck due to respiration movement, cycleGAN failed to inhibit several serious artifacts, especially at the chest wall and heart with great movements. AGGAN modified the generator of cycleGAN via the background attention mask focusing on constant areas and foreground attention masks focusing on changing areas and combined them to generate the final sCT images. The quantitative evaluation of sCT images for testing patients and a phantom demonstrated that the sCT images generated by AGGAN achieved the best image quality, with the highest SSIM, PSNR and the lowest MAE. The accuracy of dose calculation in radiotherapy is closely related to the accuracy of HU values in CT images. The statistical analysis on 3D gamma passing rates of dose distribution demonstrated that sCT image generated from all the three methods significantly improved the accuracy of dose calculation compared with original CBCT image. The sCT generated by AGGAN offered the highest gamma passing rates under the strictest criteria of 1 mm/1% compared with other methods. The sCT generated by AGGAN showed the best performance in correcting HU value of the image, the anatomical structures preservation and dose calculation in radiotherapy.

## Conclusions

Unpaired low-dose thoracic CBCT and CT images were trained by AGGAN. The generated high-quality sCT images reduced most artifacts and preserved the anatomical structures well. The sCT generated by AGGAN provided high-accuracy dose distribution calculation and can thus be applied to adaptive radiotherapy.

## Data Availability

The datasets used during the current study are available from the corresponding author on reasonable request.
